# *Ansa*–Ferrocene Derivatives as Potential Therapeutics

**DOI:** 10.3390/molecules29204903

**Published:** 2024-10-16

**Authors:** Marcin Cybulski, Olga Michalak, Włodzimierz Buchowicz, Maria Mazur

**Affiliations:** 1 Chemistry Section, Pharmacy, Cosmetic Chemistry and Biotechnology Research Group, Łukasiewicz Research Network–Industrial Chemistry Institute, Rydygiera 8, 01-793 Warsaw, Poland; marcin.cybulski@ichp.lukasiewicz.gov.pl (M.C.); olga.michalak@ichp.lukasiewicz.gov.pl (O.M.); 2 Faculty of Chemistry, Warsaw University of Technology, Noakowskiego 3, 00-664 Warsaw, Poland; wlodzimierz.buchowicz@pw.edu.pl

**Keywords:** ferrocene, *ansa*–ferrocene, ferrocenophane, biological activity, anticancer

## Abstract

It has been known since the 1990s that the introduction of a ferrocenyl–type substituent into compounds with proven biological activity can improve their properties. More recently, it was also shown that a carbon bridge connecting the two cyclopentadienyl rings in ferrocene derivatives could enhance the biological properties of the new compounds compared to those without them. However, the synthesis of ferrocenes with this additional linker, known as *ansa*–ferrocenes, is more difficult due to advanced synthetic protocols and the phenomenon of planar chirality in ring–substituted compounds. As a result, research into the formation of hybrids, conjugates and other *ansa*–ferrocene derivatives has not been widely conducted. This review discusses the potential biological properties of these units, covering scientific articles published between 1980 and 2024.

## 1. Introduction 

Due to their unique physical and chemical properties, structural diversity, ability to ligand exchange, redox and catalytic properties, organometallic compounds represent a promising group of potential drugs [1,2,3]. Among them, ferrocene and its derivatives have been used in the preliminary development of novel compounds with compelling structures and properties [4]. In the field of bioorganometallic chemistry, the ferrocenyl moiety often serves as a surrogate for functional groups [5,6], usually aromatics, in molecules with established biological activity. Novel compounds synthesized in this way display improved properties when compared to their parent molecules [7,8,9,10,11].

Maintaining a balance between reactive oxygen species (ROS) levels is essential for normal cell function [12,13]. While the presence of ROS had been previously considered detrimental by inducing oxidative stress, some studies have unexpectedly shown that antioxidants could also promote cancer formation and growth [14]. However, it is now accepted that appropriate levels of ROS interfere with normal cell signaling. To fully understand the potential of targeting ROS in cancer therapy, studies of the mechanism of action are essential. Some studies suggest that the mechanism of action of ferrocenes involves reversible redox chemistry and the generation of ROS [15,16,17]. Released ferrocene complexes can form highly reactive hydroxyl radicals, leading to the generation of ROS [18]. This disrupts the ROS balance via the Fenton pathway, leading to DNA damage and cancer cell death. It has also been shown that the rate of accumulation of ferrocenes in cancer cells is proportional to their cytotoxic activity, i.e., the mechanism responsible for cell death correlates with the transport of ferrocenes into cancer cells. The uptake of ferrocenes into cells is mediated by membrane glycoproteins called transferrin receptors. These receptors are responsible for the cellular uptake of iron from plasma. They can also transport various metal ions and organometallic compounds from the bloodstream to all tissues. This has been demonstrated in a molecular docking study [19]. Although a number of ferrocene derivatives have shown promising biological activity, the potential of *ansa*[n]–ferrocenes (also known as [n]ferrocenophanes) in medicinal chemistry was discovered not long ago. This review discusses the latest developments in research on *ansa*–ferrocenes published in the literature between 1980 and 2024. It includes an analysis of their biological properties, SAR studies and an evaluation of their mechanism of action.

## 2. Anticancer Properties

### 2.1. Ferrocenophanes Derived from Ferrocifene Derivatives

**Figure 1:**
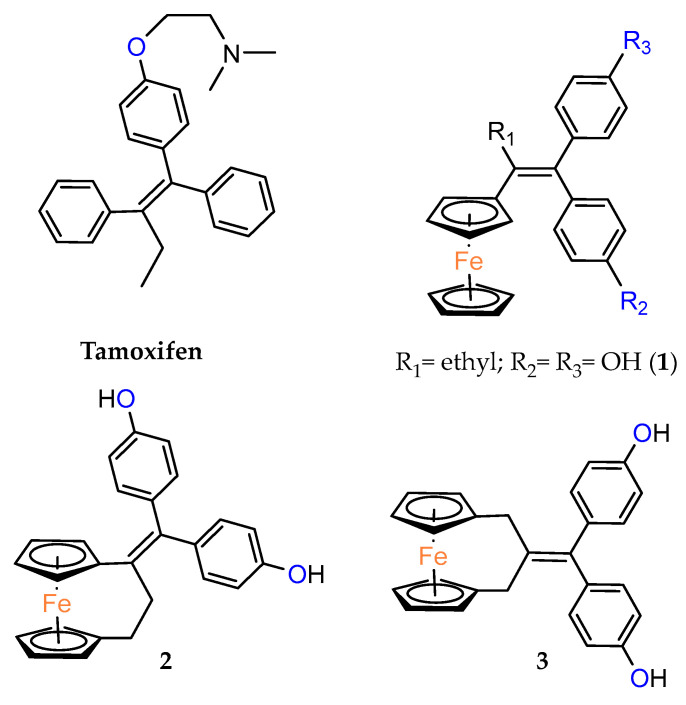
Tamoxifen, ferrocifen (**1**) and its *ansa* derivatives of ferrociphenols.

Ferrocenophane phenols are a type of ferrocene named after the first published examples of hybrid compounds containing both ferrocene fragments and the anticancer drug tamoxifen [20]. Tamoxifen is a nonsteroidal selective estrogen receptor modulator (SERM) used to treat all stages of hormone receptor–positive breast cancer [21]. Ferrocenyl hydroxytamoxifen **1** as a prototype for a new range of cytostatic agents targeting the estradiol receptor site was published in 1996 (Figure 1) [8]. Ferrocifenes were the first examples demonstrating that the modification of polyphenols with a ferrocenyl substituent can significantly increase cytotoxicity in vitro [22,23]. Although the compounds were designed to target delivery of the cytotoxic ferrocene group to cells overexpressing estrogen receptors, they showed significant in vitro activity against both hormone–dependent MC–7 cells and triple–negative MDA–MB–23 breast cancer cells (IC_50_ = 0.7 μM and IC_50_ = 0.6 for **1**, respectively). These observed antiproliferative effects were much stronger than those of 4–hydroxytamoxifen, the reference antiestrogen and ferrocene alone, which was inactive against these breast cancer lines. However, the recent cytotoxicity values for **1** have been inconsistent with those previously published [24]. The IC_50_s determined were only 43.3 μM for MCF–7 cells and 26.3 μM for MDA–MB–231 cells after 72 h of incubation. In this study, compound **1** also showed activity against PANC1 pancreatic cancer cells with an IC_50_ of 12.5 μM, twice that of tamoxifen, and significantly increased cellular oxidative stress compared to the reference compound. It is noteworthy that, while promising structures for pancreatic cancer therapy are still being investigated in the case of ferrocene derivatives (see, for example, ferronucleoside, which inhibits DNA replication in a panel of pancreatic cancer cells [25]), no such reports have been published in the case of *ansa*–ferrocenes.

Over the last three decades, intensive SAR research has led to the discovery of 300 ferrocifenes with various substitutions at the R_1_, R_2_ and R_3_ positions (see Figure 1). Some of these compounds have demonstrated potent anticancer properties against various MDR cancer cell lines, including glioma, metastatic melanoma, breast cancer and leukemia [26]. One direction of synthetic modifications to improve the biological activity of ferrociphenols has been the introduction of carbon, or other atoms containing bridges, linking the two cyclopentadienyl rings in the structure.

Jaouen and coworkers, the leading research group in the synthesis and biological evaluation of ferrocifen analogues, first demonstrated the effect of stiffening structure **1** on biological activity. Two rigid analogues **2** and **3** with cyclopentadienyl rings connected by a three–carbon bridge were designed, synthesized and compared in bioassays with a flexible analogue [27]. Studies of relative binding affinity (RBA) for the estrogen receptor (ER) and activity against breast cancer lines MCF–7 ER+ and MDA–MB–231 ER– and PC–3 prostate cancer cells showed the greatest difference in cytotoxicity of the compounds on hormone–independent MDA–MB–231 and PC–3 cells. (Table 1) Rigid compound **2** was one order of magnitude more cytotoxic than flexible compound **1** or rigid **3,** which lacks conjugation between the phenolic and ferrocenyl groups. Both rigid compounds had estimated cytotoxic activity comparable to **1** for MCF–7 cells. These results suggested that there may be a competition between the positive estrogenic effects and negative cytotoxic effects on this cell line, which could not be predicted from the simple binding values. Compound **4**, with one hydroxyl group, **5** with one amine group in the phenyl ring and **6** with an acetamide substituent showed similar activity against MDA–MB–231, acting less effectively than analogue **2** [28]. Replacement of one hydroxyl in **2** by amine (**8**) or acetamide (**10**) substituents resulted in similarly high activities against MDA–MB–231 with IC_50_ = 0.06 and 0.09 μM, respectively. Compound **11** with one amine group in each phenyl ring also showed high activity. It was in contrast to the result for its acetamide analogue **9**, which acted two orders of magnitude weaker. Interestingly, acetylated prodrug analogues of **2,** i.e., mono acetylated **12** or peracetylated **13**, acted at the level of the leading structure against MD–MB–231 [29,30].

Compound **5** in extended screening in vitro revealed better activity against human glioblastoma SF–295 (IC_50_ = 1.0 µM), human ileocecal colorectal adenocarcinoma HCT–8 (IC_50_ = 0.5 µM) and human promyelocytic leukemia HL–60 (IC_50_ < 0.12 µM) than against melanoma cancer line MDA–MB–435 (IC_50_ > 61.68 µM) derived from the M14 line [32]. Moreover, it showed promising characteristics possessing of high antiproliferative activity and low hemolytic activity on mouse erythrocytes [33].

Further study indicated that hybrids **2**, **8**, **10** and **12** could act as DNA alkylating agents or DNA antimetabolites [11,29]. Particularly, hybrid **2** (IC_50_ range 48–580 nM) possessed a broad activity spectrum against a panel of 60 human cancer cell lines, derived from nine different cancer types: leukemia, lung, colon, CNS, melanoma, ovarian, renal, prostate and breast [29,30]. The highest sensitivity of **2** was observed in vitro on human SK–Mel28 melanoma cells (IC_50_ = 1.2 μM) [26,34]. Compound **2** was also tested on three ovarian epithelial cancer cell lines, including A2780–Cis resistant to cisplatin (IC_50_ = 0.359 µM for A2780; IC_50_ = 0.165 µM for A2780–Cis; IC_50_ = 1.910 µM for SK–OV–3). However, **2** showed a twofold higher selectivity factor (SF = 0.46) against A2780 than the cisplatin–resistant line, when compared to normal human pulmonary fibroblasts MRC5 [35]. The hybrid **2** also showed acceptable acute toxicity in mice, with the maximum tolerated dose of 100 mg/kg. Compound **2** induces senescence in various cancer cell models associated with distinct sensitivity to pro–apoptotic stimuli [36]. The high and broad–spectrum anticancer activity as well as low toxicity made this hybrid a useful starting point in drug development to combat various types of cancers.

Another study was conducted to investigate the impact of the length of the carbon bridge connecting the cyclopentadienyl rings on the activity against MDA–MB–231 cells [37]. The analogues of compound **2** with a four– (**19**) and five–carbon bridge (**20**) exhibited an order of magnitude lower activity than the original structure of **2**. Furthermore, the introduction of an additional methyl substituent into the compound altered its activity. However, the R–isomer (**21**) demonstrated activity similar to that of **2**. The other compound of interest was the relatively bulky derivative of [5]ferrocenophane with two –[bis–(4–hydroxyphenyl)]methylidene groups (**24**). This relatively large compound was still active with an IC_50_ of 2.7 μM [37].

The anticancer activity of hybrid derivatives of superoylanilidine hydroxamic acid (SAHA) having a [3]ferrocenophan–1–ylidene substituent was also evidenced [38]. The compounds **25**, **26** and **27** showed strong antiproliferative activity against triple–negative MDA–MB–231 cells, with IC_50_ values in the 0.84–2.72 μM range. The primary amide **26** was found to be slightly more cytotoxic than its hydroxamide analogue **27**. The cytotoxic effects of compounds **25–27** were also observed on hormone–dependent MCF–7 breast cancer cells (Table 2), where all three compounds (**25**, **26** and **27**) showed antiproliferative activity with IC_50_ values in the range of 0.87–4.05 μM. In contrary, their analogues lacking the organometallic moiety were unable to inhibit 50% of cell growth even at concentrations of 10 μM. The exception was SAHA (IC_50_ = 1.04 μM), although ferrocenophane hydroxamide **27** (IC_50_ = 0.87 μM) was again slightly more active. In general, incorporation of the ferrocenophane and ferrocene derivatives of hydroxytamoxifene into the suberamide structure enhanced the antiproliferative activity of the resulting compounds against MDA–MB–231 and MCF–7 cancer lines. Of the compounds tested, ferrocenophane derivatives showed the greatest cytotoxicity on MCF–7 breast cancer cells. The electrochemical behavior of these ferrocenophanic suberamides suggested that they undergo redox activation, which may contribute to their antiproliferative activity [38].

**Figure 2:**
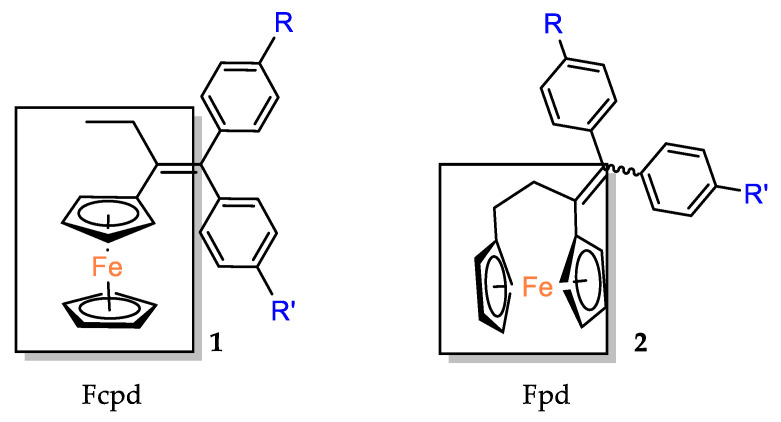
The 1–ferrocenylpropylidene group (Fcpd) and [3]ferrocenophan–1–ylidene group (Fpd).

The cytotoxic activity of the [3]ferrocenophan–1–ylidene (Fpd) bis–aniline 11 (Fpd, Figure 2) analogues, additionally substituted at the amine groups, was evaluated against hormone–resistant MDA–MB–231 breast cancer cells [39]. The straight dose–dependent effects were not observed for compounds **28**, **29** and **30**. The percentage of cell growth inhibition at concentrations of 1 μM was found to be 27%, 23% and 18%, respectively. The decrease in activity observed for the analogues was accompanied by an increase in the length of the acyl carbon chains. At concentrations one order of magnitude higher (i.e., 10 μM), only compound **29** inhibited the growth of the cancer cell line by more than 50%, reaching 51%. The compounds obtained demonstrated reduced activity compared to the simple diacetanilide derivative **9** (IC_50_ = 5.64 μM). Furthermore, it was demonstrated that modifying the substituents from a basic amine to an amide distinctly diminished the activity against MDA–MB–231 cells. All anilides obtained demonstrated a minimum of two orders of magnitude lower cell growth inhibition when compared to bis–aniline **11** (IC_50_ = 0.05 μM) [39]. The activation mechanism was found to be linked to the intramolecular electron transfer process previously observed for phenolic derivatives. The conversion of Fcpd compounds to ferrocene has been postulated to promote an increase in the ROS level, which can result in direct DNA damage and/or the activation of tumor suppressor genes, leading to apoptosis or senescence of cancer cells [39].

Replacement of the OH group in **2** by two dimethylaminoalkyloxy chains decreased the cytotoxicity effect on MDA–MB–231 in compound **31** [40]. Against MCF–7 cells, compound **31** showed a slight, but reproducible, proliferative estrogenic effect at low concentrations (1 × 10^−8^ and 1 × 10^−7^ M). Following observation on MDA–MB–231 cells, it was very cytotoxic against MCF–7 at higher concentrations (between 1 × 10^−7^ M and 1 × 10^−6^ M). According to the binding affinity biochemical studies, **31** appeared highly recognized by the alpha form of ER. While the ability to generate quinone methide formation was blocked in the structure of **31**, an alternative mechanism of its cytotoxicity was investigated in in silico studies. The estimated affinity of the compound to Zn^2+^ and Ca^2+^ indicated the complexing role of two aminoalkyl chains on these cations as a potential mechanism of action [40].

The introduction of an additional hydroxyl group in the *meta* position of the *para*–hydroxyphenyl ring in compound **4** led to a compound **32** with similar biological activity to that of compounds **4**, **6** and **31** (MDA–MB–231 breast cancer cells). The high antiproliferative activity of compound **31** was attributed to the narrower HOMO–LUMO gap present in the oxidized form of [3]ferrocenophane moieties, resulting in more reactive species [41].

A new series of compounds with various aminoalkyloxy side–chains (O(CH_2_)_3_NMe_2_, O(CH_2_)_3_piperidine, O(CH_2_)_3_–pyrrolidine, NHCO(CH_2_)_2_NMe_2_) were also characterized in terms of their antiproliferative activity against the MDA–MB–231 cancer cells. The IC_50_ values of compounds **33**, **34** and **36** were between 0.17 and 0.19 μM. These values were twice as weak as for the model diphenol **2** (0.09 μM). Nevertheless, these derivatives were among the most efficacious compounds against MDA–MB–23 ever obtained. The lipophilicity of these compounds did not appear to play an important role in their activity. Once again, it was demonstrated that systems lacking an OH group, as in **35** and **39**, are less active. Although, the substitution of the aminoalkoxy chain –O(CH_2_)_3_NMe_2_ by the amido chain NHCO(CH_2_)_2_NMe_2_ may produce different effects, the IC_50_ values of compounds **35** and **39** were very similar. In contrast, amido compound **38** exhibited three times better cytotoxicity than **33** (IC_50_ = 0.05 μM vs. 0.18 μM), belonging to the most active *ansa*–derivatives ever studied against the MDA–MB–231 cancer cell line. For comparison, molecules **26** and **27** were slightly less effective than compounds **35** and **39**. The lengthening of the amido chain may impact their diminished activity [42]. The in vitro effect of the new complexes **26**, **27** and **33–39** on the growth of MCF–7 hormone–dependent cells was then studied concerning their estrogenic and antiestrogenic properties. The antiestrogenic effect was not observed in the ferrocenophane series **26**, **27** and **33–39**, although a slight estrogenic effect was observed for compounds **33**, **34, 38** and **39**. The compounds, in general, preserved an affinity for the estrogen receptor. Thus, the estrogenic or antiestrogenic effects were then analyzed exhaustively on the MCF–7 cells for two compounds, **33** and **40**, which differed only in the length of the aminoalkoxy chain. It was investigated at three concentrations (1, 10, 100 nM). Compound **33** triggered an estrogenic effect at 1 nM, which was inverted at a higher concentration of 100 nM due to the appearance of a cytotoxic effect. In contrast, for compound **40**, the antiestrogenic effect started immediately from 1 nM. Therefore, the authors proposed that this phenomenon could be attributed to the length of the aminoalkyl side chain, which may change the estrogenic effect into an antiestrogenic, as was confirmed in the modeling study [42].

Furthermore, the same research group also studied the MDA–MB–231 cell line inhibition growth produced by compounds slightly more different in structure than typical ferrocenophane phenol derivatives (Table 3) [42]. Compounds **48** and **49**, containing a 1,2–diol moiety in the place of a double bond present in model compound **2**, showed the best antitumor properties in the obtained group. The results were comparable to these for their analogues **4** and **7**, i.e., at the 0.1–0.01 μM level of IC_50_. Moreover, it is interesting to note that two diastereoisomers **49** and **50** differ slightly in their activities.

### 2.2. Other Ferrocenophanes with All Carbon Bridges

**Figure 3:**
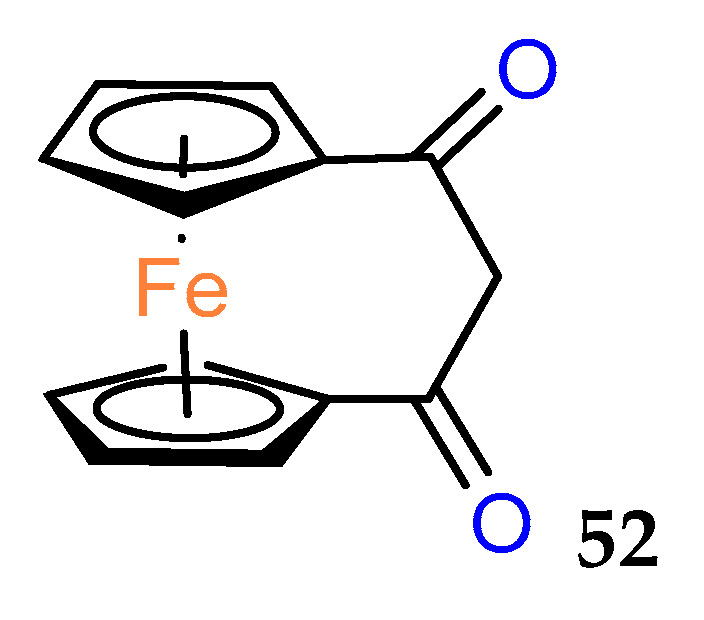
[3]Ferrocenophane–1,3–dione.

The history of biologically active ferrocenophanes is mainly based on the ferrociphenol derivatives. However, as early as 1980, a simple [3]ferrocenophane–1,3–dione **52** (Figure 3) was tested in vivo against epithelial carcinoma and was found to be inactive [43].

Another interesting group of compounds was designed to enhance the activity of the antibiotic platensimycin. Although the obtained *ansa*–ferrocene derivatives did not show antimicrobial properties, their intermediates **54** and **55** with protective groups on carboxylic substituents and alternatively on hydroxyl substituents showed in vitro anticancer activity (Table 4). In particular, compound **54**, in which only the carboxyl group was protected as a 2–(trimethylsilyl)ethyl (TMSE) ester, showed activity at the micromolar level against pancreatic ductal adenocarcinoma PT45 and hepatocellular carcinoma HEPG2 [44].

Recently, Yan et al. combined [3]ferrocenophanes and 2–aza–[3]ferrocenophanes **56–64** with benzohydroxamic acid, via a piperazine or piperidine linker, to obtain a new series of ferrocenyl hydroxamic acids (Table 5). Two *ansa*–ferrocene hybrids have shown inhibition of histone deacetylase 6 (HDAC6) at nanomolar concentrations, and this enzyme has been considered a possible target for anticancer and antineurodegenerative therapies [45]. Selected effective inhibitors of HDAC6 were further tested on other HDAC subtypes to identify compound **60** as the most selective to HDCA6 with selectivity ratios, to others from the HDCA family, ranging from 13 to more than 261. Both HDAC6 inhibitors **60** and **63** revealed moderate antiproliferative activity against a panel of cancer cell lines including a prostate cancer cell line (22RV1), an immunoglobulin λ myeloma cell line (MM1.S), a monocyte leukemia cell line (MV4–11), a mantle cell lymphoma cell line (JEKO–1) and a breast cancer cell line (4T1). Compound **63** also induced apoptosis in 4T1 cancer cells in a dose–dependent manner. Western blot studies showed altered levels of proteins involved in the apoptosis pathway under the influence of compound **63**, e.g., PARP or caspase–3. The possible synergism of the pro–apoptotic effects of the compounds was also pointed out by the fact that HDAC inhibitors have the potential to induce apoptosis spontaneously and ferrocene complexes can induce apoptosis through ROS. Thus, this was confirmed by showing an increase in total ROS in 4T1 cancer cells after incubation with **63**.

Buchowicz and coworkers designed and synthesized new uracil–triazole–[4]ferrocenophane hybrids with antitumor activity against breast and lung cancers. The antitumor potential of *ansa*–ferrocene (±)–**65** was evaluated on three cell lines: the hormone–dependent breast cancer cell line MCF–7, the triple–negative breast cancer line MDA–MB–231 and A549 lung cancer cells (Table 6). Compound (±)–**65** acted at the cisplatin level against all cancer lines studied and showed better activity than its nonbridged analogue. The compound also showed significantly lower toxicity against normal MRC–5 cells than cisplatin, particularly in the case of lung cancer A549, where SI for (±)–**65** was 4.2 vs. 1.8 for cisplatin [46].

The same research group published a synthesis and biological evaluation of (allylaminomethyl) *ansa*–ferrocene derivatives (±)–**66** and (±)–**67**, which were then compared with their ferrocene analogues [47]. The anticancer effects of (±)–**66** and (±)–**67** were also evaluated against hormone–dependent MCF–7 breast cancer and A–549 lung cancer cells. Furthermore, the compounds were studied using prostate adenocarcinoma PC–3 and mouse noncancerous fibroblasts line Balb 3T3. The tested cell lines showed twice as much sensitivity to *ansa* analogues as their ferrocene counterparts (the same order of magnitude). Both compounds demonstrated the highest response against MC–7 cells. Compound (±)–**67** was, therefore, administered to examine the effect on the cell cycle progression in this cancer line. The studies showed cycle arrest in the S–phase of DNA replication following administration of increasing concentrations of (±)–**67**. In addition, a sub–G1 phase was observed in MCF–7 cells treated with 40 µM and 60 µM of compound (±)–**67**, indicating the apoptotic nature of an action of (±)–**67**.

### 2.3. Ferrocenophanes with Bridges Containing Heteroatoms

The biological properties of ferrocenophanes have been tested and demonstrated in several compounds. In addition to the most extensively studied carbon–bridged compounds, the other examples of *ansa*–ferrocenes contain nitrogen (e.g., **63**, **64**), selenium or phosphorus. We note that research on the selenium–bridged compounds is a significant contribution to the broader studies on the bioactivity of different organoselenium derivatives related to their antioxidant and anticancer properties [48].

**Figure 4:**
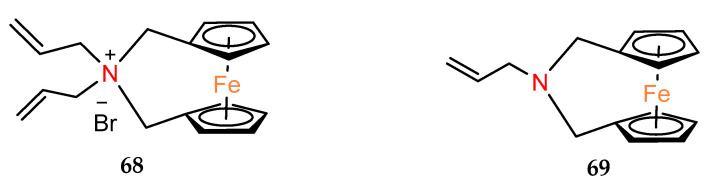
Compounds **68** and **69**.

In studies on allyl derivatives of ferrocene [47], the cytotoxicity of *ansa*–ferrocenes with nitrogen in the bridge was also checked. Compounds **68** and **69** (Figure 4) showed weak activity (EC_50_ > 100 µM) for each of the tested cell lines.

In studies on the cytotoxicity of organoselenium dopamine conjugates, the *ansa*–ferrocene derivative **70** was found to be the most active in the cytotoxicity assays using AGS (gastric adenocarcinoma), A2780 (ovarian carcinoma), A549 (non–small–cell lung carcinoma), BxPC–3 (pancreatic cancer), HepG2 (hepatocellular carcinoma) and MGC–803 (human gastric cancer) cell lines (Table 7) [49]. HepG2 appeared to be the most sensitive line, with an IC_50_ value of 2.2 ± 0.5 μM. Therefore, the cell cycle distribution in HepG2 was analyzed after administration of 10 or 20 µM of the compound. G1 arrest was observed by indicating a dose–dependent increase in the population of cells in this phase. Subsequently, the population of cells in the S–phase decreased significantly. A dose–dependent presence of cells in the G2/M phase was also observed. Because cell cycle arrest plays a role in apoptosis, the dose–dependent ability of the tested compound to induce apoptosis or necrosis in HepG2 cells was further confirmed by flow cytometry in the presence of annexin stain. The late–stage apoptosis rates of HepG2 cells treated with 10 and 20 µM increased in a dose–dependent manner to 13.09% and 33.62%, respectively. The necrotic rate of HepG2 cells also increased. As a result, compound **70** was effective in directly killing cells or inhibiting the cells growth. Biochemical tests also showed an increase in the pro–apoptotic enzymes caspase 3 and caspase 9. The expression of the pro–apoptotic protein Bax also increased, while a decrease in the anti–apoptotic protein Bcl–2 was observed. Immunoblotting studies also showed an increase in the expression level of the p53 suppressor protein after administration of compound **70**. Encouraging results from in vitro tests led the authors to in vivo experiments, where compound **70** was shown to inhibit tumor growth in nude mice bearing HepG2 tumor xenografts. In addition, **70** inhibited both tubule formation and endothelial cell (HUVEC) migration in the anti–vascular activity assay.

Based on the promising results for the seleno–organic derivative of dopamine **70**, a similar dopamine, aza–*ansa*–ferrocene **71,** was obtained among a novel group of compounds. The mechanistic study revealed that the cytotoxicity of these ferrocenyl seleno–dopamine derivatives was mainly related to the Fenton–like reaction under physiological conditions [50].

Core–shell conversion nanoparticles (UCNPs) were functionalized via surface coordination chemistry protocols using *ansa*–ferrocene derivatives **70** and **71** to give conjugates Fc–UCNPs [51]. A drug carrier form (Fc–UCNP) was also obtained by encapsulating Fc–UCNPs in liposomes. It was assumed that the obtained conjugates would be able to release OH radicals in a Fenton–photo reaction activated by NIR radiation to induce cancer cell death. In vitro studies on AGS and MGC–803 cancer lines evidenced the radiation effect in compound **72** (Table 7). None of the two *ansa*–ferrocene derivatives, **72** nor **73**, was selected for further in vitro studies, which demonstrated that the UCNP–Lipo model conjugate, in a xenograft model using AGS cancer cells in BALB/c nude mice, underwent preferential accumulation in a tumor site followed by its enhanced uptake to cancer cells.

**Figure 5:**
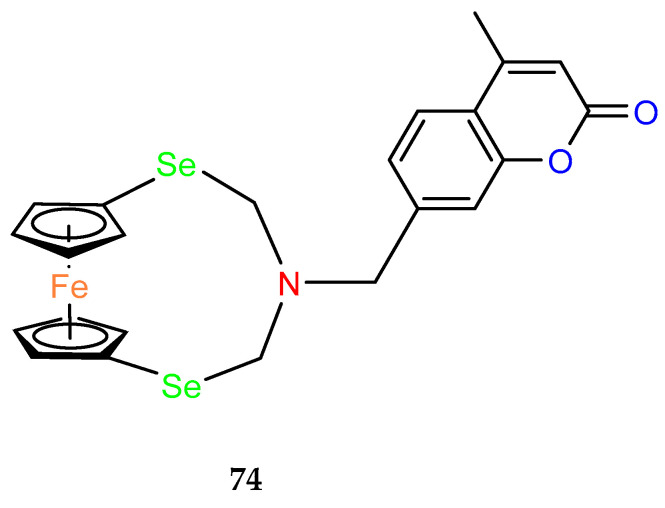
Compound 74.

To investigate the utility of *ansa–*ferrocene derivatives as compounds with phototriggering properties, a noncovalent complex containing an Eu^3+^ cation and a ClO_4_^–^ anion in the structure of a ferrocenophane derivative with coumarin fluorophore **74** was prepared (Figure 5). In in vitro cell line studies, the compound, which exhibits photoluminescence upon UV irradiation ≥ 365 nm, showed phototriggered anticancer activity against HepG2 liver cancer cells, as confirmed by confocal imaging [52].

Five ferrocene chloroquine derivatives with the terminal nitrogen of the chloroquine derivative bridging the two cyclopentadienyl rings of ferrocene (**75–79**) were synthesized by Salas et al. [53]. They have been characterized in terms of antiplasmodial activity, as well as cytotoxicity on breast epithelial cells cell line MCF–10A, commonly used as a model for normal human breast cells [54] and melanoma cancer line MDA–MB–435S (but not a breast cancer cell line [55] as mentioned in the cited paper [45]) (Table 8). All tested compounds were found to lower the viability of the cancer cell culture but with low selectivity toward normal cells. Of the series studied, compound **79** appears to be the most active and with the worst selectivity index versus normal line SI = 0.5.

Ferrocenophane with a bridge of two phosphorus atoms coordinated to gold(I) was tested in vitro on various cancer cell lines in the 1990s [56]. Compound **80** showed the best activity against the SW1116 cell line (IC_50_ = 48.9 µg mL^–l^). The cytotoxicity, although improved by the presence of gold, was not better than the cytotoxicity of the well–known drug cisplatin (Table 9).

## 3. Miscellaneous Biological Activities Properties of *Ansa*–Ferrocenes

*Ansa–*ferrocenes **2** and **7** were also investigated as potential blockers of melanin production, for use in cosmetics as compounds that reduce skin pigmentation by acting through a tyrosinase inhibition mechanism [57]. Compound **7**, out of the 20 compounds tested, showed significant inhibition of one of the reactions catalyzed by tyrosinase, i.e., hydroxylation of monophenol (monophenolase activity), by more than 50% at a concentration of 20 μM. In biochemical studies, *ansa–*ferrocene **7** was also classified as a reversible tyrosinase inhibitor.

The antiplasmodial activities of compounds **75–79** were evaluated in vitro against the chloroquine–sensitive (CQS) D10 and the chloroquine–resistant (CQR) Dd2 and K1 strains of *P. falciparum* (Table 10). All compounds showed activity against all the tested parasite strains [53]The most active compound of this group is **77** with IC_50_ values of 91.3 and 152.2 nM in the parasite strains D10 and Dd2, respectively.

Antifungal properties of thia– and aza–ferrocenophanes were also studied. The most promising derivative in this series was found to be the aza–compound **82**, which acted in vitro with an inhibition ratio of 100% for *B. cinerea* and 88.5% for *P. piricola* in the 50 mg/mL concentration (Table 11) [58].

The antibacterial activity of the [3]ferrocenophane phenol analogue (**31**) was studied by el Arbi et al. [59]. Compound **31** was converted to citrate salt to ensure its greater solubility, and then tested against various Gram–positive and Gram–negative foodborne pathogen strains (Table 12). Citrate of **31** was as active as its ferrocenyl counterpart but displayed a twice lower therapeutic index of 0.5 with an HC50 hemolytic activity of 22.4 μM. A [3]ferrocenophane with a sulfur–only bridge has been tested for antimicrobial activity. A variety of bacteria and fungi were used for this study. Compound **85** showed moderate activity (Table 12) [60].

For model compound **2**, which was very active against MDA–MB–231 cancer cells (see the Anticancer Properties section), the evaluation of the antimicrobial activity on the bacteria *Pseudomonas aeruginosa* and *Staphylococcus aureus*, as well as the fungus *Candida albicans* was performed. As a result, no correlation between the antimicrobial, antifungal and anticancer activity was found for **2**. The compound appeared to be inactive against the microorganisms tested [61].

## 4. Conclusions

Similarly to ferrocene derivatives, ferrocenophanes have attracted interest as compounds with potential anticancer, antibacterial and antifungal properties. The best–known derivative of this group is [3]ferrocenophane phenol **2**, a bridged analogue of flexible ferrocifen **1**. Summarizing, among compounds presented in this review, 61 analogues belong to the [3]ferrocenophane family, thus this structure can be considered the most promising lead for new drug development. However, due to the more complicated synthesis, conformational constraints affecting the outcome of chemical reactions [62], the isolation and purification methods or the phenomenon of planar chirality [63], fewer *ansa*–ferrocene derivatives have been tested so far, at least in preliminary biological tests, when compared to their unbridged analogues. Some reports point to nonscientific problems related to the increasing difficulty of obtaining funding for the development of metallocenes as drugs, including bureaucratic restrictions [64]. Therefore, it appears that compounds from this group will not easily enter the phase of intensive clinical trials, especially since neither of the two well–known ferrocene derivatives, ferroquine and ferrocifen, have found their way onto the drug market. However, the antimalarial potential of ferroquine is still exploited through various clinical trials testing this compound in combination therapies. For example, the recent contribution of artefenomel to the clinical and parasiticidal activity of ferroquine and artefenomel in uncomplicated Plasmodium falciparum malaria has been investigated [65]. Moreover, the Zydus company applied for a phase I clinical trial of ferroquine and novel triaminopyrimidine ZY–19489 combined therapy against malaria [66].

## Figures and Tables

**Table 1:** Relative binding affinity (RBA) for the estrogen receptor * [27,31].

Compound	RBA (%)	
	ERα	ERβ
**1**	9.6 ± 0.9	16.3 ± 1.5
**2**	7.2 ± 0.7	4.84 ± 0.4
**3**	7.6 ± 0.6	15.4 ± 0.4
**31**	2.05 ± 0.08	–
**33**	17.2	–
**34**	14.4	–
**35**	4.8	–
**38**	10.3	–
**39**	4.4	–

* see structure of compounds in Table 2.

**Table 2:** Cytotoxicity against breast and prostate cancer lines.

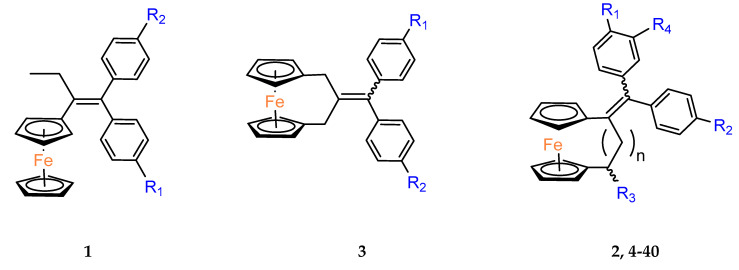									
Compound	Ref.	n	R_1_	R_2_	R_3_	R_4_		IC_50_ (μM) *	
							MCF–7	MDA–MB–231	PC–3
**1**	[22]	–	OH	OH	–	–		0.64 ± 0.06	0.7 (1 exp)
**2**	[27]	1	OH	OH	H	H	4	0.09 ± 0.01	0.094 ± 0.006 or 0.14 ± 0.01 [26]
**3**	[27]	–	OH	OH	–	–	1	0.96 ± 0.03	1.080.02
**4**	[28]	1	H	OH	H	H	–	0.47 ± 0.01	–
**5**	[28]	1	H	NH_2_	H	H	–	0.21 ± 0.03	–
**6**	[28]	1	H	NH–Ac	H	H	–	0.47 ± 0.04	–
**7**	[29]	1	H	H	H	H		0.92 ± 0.11	2.43 ± 0.47 [26]
**8**	[29]	1	NH_2_	OH	H	H		0.061 ± 0.005	0.03 ± 0.01 [27]
**9**	[29]	1	NH–Ac	NH–Ac	H	H		5.64 ± 1.13	12.45 ± 0.85 [27]
**10**	[29]	1	NH–Ac	OH	H	H		0.092 ± 0.019	0.02 ± 0.00 [27]
**11**	[29]	1	NH_2_	NH_2_	H	H		0.047 ± 0.010	0.05 ± 0.00 [27]
**12**	[29]	1	OAc	OH	H	H		0.049 ± 0.003	
**13**	[29]	1	OAc	OAc	H	H		0.044 ± 0.001	
**14**	[29]	1	OAc	H	H	H		0.260 ± 0.004	
**15**	[29]	1	Br	H	H	H		2.93 ± 0.62	
**16**	[29]	1	Br	Br	H	H		>10	
**17**	[29]	1	CN	H	H	H		0.85 ± 0.07	
**18**	[29]	1	CN	CN	H	H		7.98 ± 1.20	
**19**	[37]	2	OH	OH	H	H		2.41 ± 0.10	
**20**	[37]	3	OH	OH	H	H		1.85 ± 0.28	
***R*–21**	[37]	1	OH	OH	CH_3_	H		0.78 ± 0.12	
***S*–21**	[37]	1	OH	OH	CH_3_	H		2.70 ± 0.03	
**22**	[37]	2	OH	H	H	H		4.53 ± 0.62	
**23**	[37]	3	OH	H	H	H		4.13 ± 0.18	
**24**	[37]	3	OH	OH	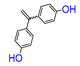	H		2.70 ± 0.30	
**25**	[38]	1	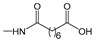	H	H	H	4.05 ± 0.57	2.72 ± 0.29	
**26**	[38]	1	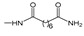	H	H	H	1.78 ± 0.40	0.84 ± 0.28	
**27**	[38]	1	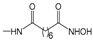	H	H	H	0.87 ± 0.14	0.94 ± 0.08	
**28**	[39]	1	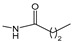	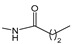	H	H		27% **	
**29**	[39]	1	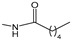	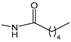	H	H		23% **	
**30**	[39]	1	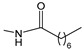	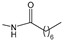	H	H		18% **	
**31**	[40]	1	O(CH_2_)_3_NMe_2_	O(CH_2_)_3_NMe_2_	H	H		0.40 ± 0.02	
**32**	[41]	1	OH	H	H	OH		0.48 ± 0.04	
**33**	[42]	1	O(CH_2_)_3_NMe_2_	OH	H	H		0.18 ± 0.04	
**34**	[42]	1	O(CH_2_)_3_NMe_2_	NH2	H	H		0.17 ± 0.02	
**35**	[42]	1	O(CH_2_)_3_NMe_2_	H	H	H		0.37 ± 0.11	
**36**	[42]	1	O(CH_2_)_3_pyr	OH	H	H		0.19 ± 0.05	
**37**	[42]	1	O(CH_2_)_3_pip	OH	H	H		1.10 ± 0.11	
**38**	[42]	1	NHCO(CH_2_)_2_NMe_2_	OH	H	H		0.05 ± 0.02	
**39**	[42]	1	NHCO(CH_2_)_2_NMe_2_	H	H	H		0.39 ± 0.03	
**40**	[42]	1	O(CH_2_)_4_NMe_2_	OH	H	H		0.12 ± 0.06	

* methylene blue assay; after 5 days of incubation, ** percent of cell growth inhibition at 1 mM; pyr = pyridine, pip = piperidine.

**Table 3:** Cytotoxicity against the MDA–MB–231 breast cancer line [31].

Compound	Structure	R_1_	R_2_	IC_50_ (μM)
				MDA–MB–231
**41**	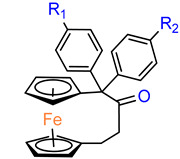	OH	OH	12.5 ± 0.3
**42**		H	OH	1.45 ± 0.49
**43**		H	H	20.1 ± 5.4
**44**		NH_2_	NH_2_	15.4 ± 0.2
**45**		NHAc	NHAc	9.38 ± 1.82
**46**	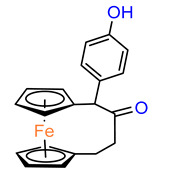	No effect at 10^−5^ M		
**47**	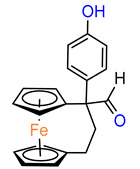	2.83 ± 0.65		
**48**	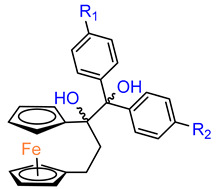	H	H	0.17 ± 0.01
**49**		H	OH	0.06 ± 0.01
**50**		H	OH	0.14 ± 0.01
**51**	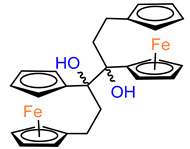	3.48 ± 0.83		

**Table 4:** Cytotoxicity of platensimycin derivatives against cancer cell lines [44].

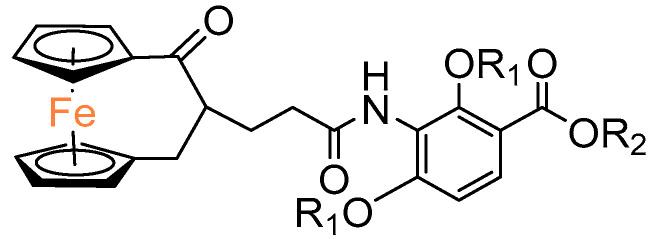				
Compound	R_1_	R_2_	IC_50_ (µM)	
			HepG2	PT45
**53**	H	H	na	na
**54**	H	TMSE	3.6 ± 0.2	2.3 ± 0.2
**55**	Me	Me	48.2 ± 5.8	55.5 ± 2.8

na: no cytotoxic activity up to 200 μM.

**Table 5:** Biological evaluation of compounds **56–64** [45].

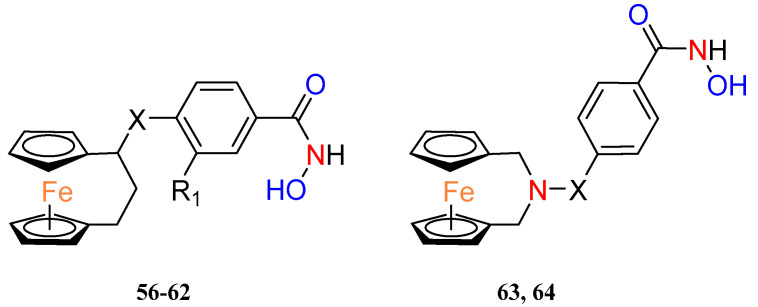								
Compound	X	R_1_	Inhibition against HDAC6 (IC_50_ (nM))	Antitumor Activities IC_50_ (µM)				
				22RV1	MM1.S	MV4–11	JEKO–1	4T1
**56**	O	H	545 ± 61					
**57**	NHCH_2_	H	534 ± 81					
**58**	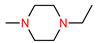	H	200 ± 20					
**59**	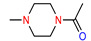	H	635 ± 54					
**60**	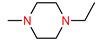	F	38.2 ± 2.1	8.90 ± 2.13	11.90 ± 2.03	7.83 ± 1.62	4.8 0 ± 1.24	16.51 ± 5.13
**61**	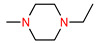	Cl	573 ± 140					
**62**	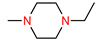	OCH_3_	2600 ± 376					
**63**	CH_2_	–	76.5 ± 10	8.54 ± 0.50	20.64 ± 4.25	7.73 ± 3.02	10.42 ± 1.98	8.95 ± 1.62
**64**	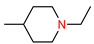	–	436 ± 130					

**Table 6:** Cytotoxicity against cancer cell lines [46,47].

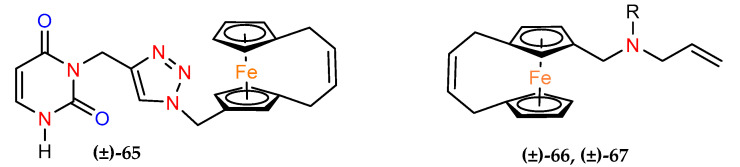						
Compound	R		EC_50_ (µM)			
		MCR–5	MCF–7	A–549	PC–3	MDA–MB–231
(±)–**65**	–	45.9 ± 1.5	28.7 ± 2.0	10.9 ± 1.7	–	30.3 ± 4.3
(±)–**66**	H		23 ± 1	71 ± 15	44 ± 13	–
(±)–**67**	CH_3_		26 ± 1	68 ± 6	76 ± 15	–

**Table 7:** Antitumor activities of ferrocenophanes with selenium in the bridge.

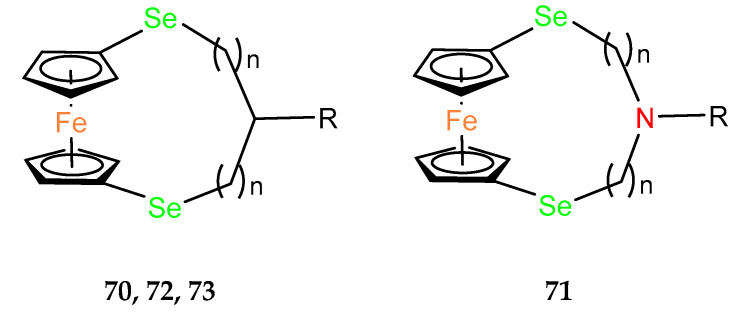								
Compound	R	n	Antitumor Activities IC_50_ (µM)					
			AGS	A2780	A549	BX–PC3	HepG2	MGC–803
**70**	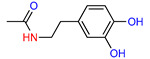	1	2.4 ± 0.4	2.3 ± 0.3	4.8 ± 1.2	5.4 ± 0.7	2.2 ± 0.5	4.5 ± 0.1
**71**	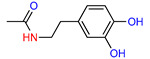	2	13.2 ± 0.9	21.1 ± 0.7			11.7 ± 0.6	7.8 ± 0.9
**72**	COOH	1	48.83 ± 2.08 * 99.74 ± 1.06					51.82 ± 3.99 * 91.55 ± 4.15
**73**	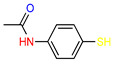	1	220.6 ± 8.7 * 243.1 ± 6.6					243.1 ± 11.0 * 244.4 ± 9.3

* with NIR (near–infrared) light radiation.

**Table 8:** Antitumor activities of ferrocenophanes **75–79**.

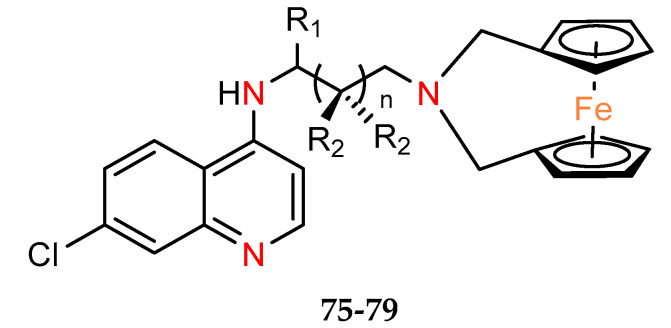					
Compound	n	R_1_	R_2_	IC_50_ (µM)	
				MCF–10A	MDA–MB–435S
**75**	0	H	–	2.4 ± 0.2	1.7 ± 0.1
**76**	1	H	H	4.0 ± 1.0	3.1 ± 0.3
**77**	2	H	H	4.2 ± 0.9	2.8 ± 0.2
**78**	0	Me	–	1.4 ± 0.1	1.4 ± 0.1
**79**	1	H	Me	0.3 ± 0.1	0.6 ± 0.1

**Table 9:** Cytotoxicity of complex **80**.

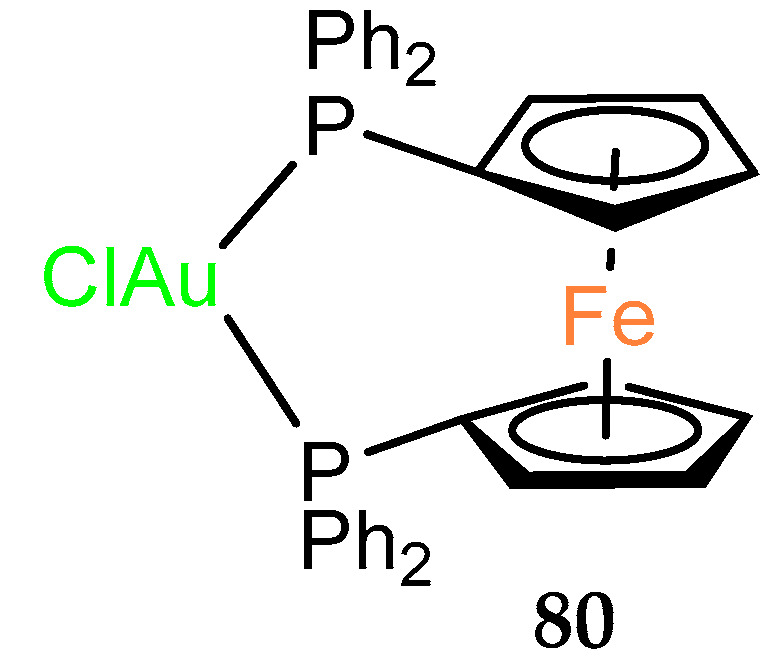							
Compound	IC_50_ (µg mL–l)						
	SW620	SW1116	ZR75–1	HT1376	SKOV–3	PA–1	LS174T
**80**	61.5	48.9	155.7	57.6	66.4	129.0	124.6

**Table 10:** In vitro antiplasmodial activity and resistance indices (RI) against *P. falciparum* CQS D10, CQR Dd2 and CQR K1 strains [49].

Compound	IC_50_ (nM)			RI	
	CQS D10	CQR Dd2	CQR K1	IC_50_ (Dd2)/IC_50_ (D10)	IC_50_ (K1)/IC_50_ (D10)
**75**	176.0	129.7 ± 23.2	ND	0.7	
**76**	323.0	224.3 ± 6.73	307.3 ± 170.5	0.7	0.9
**77**	91.3	152.2 ± 6.52	ND	1.6	
**78**	444.2	269.2 ± 4.	291.6 ± 2.24	0.6	0.7
**79**	669.0	506.5 ± 21.	ND	0.7	

**Table 11:** The fungicidal activities of thia– and aza–ferrocenophanes [58].

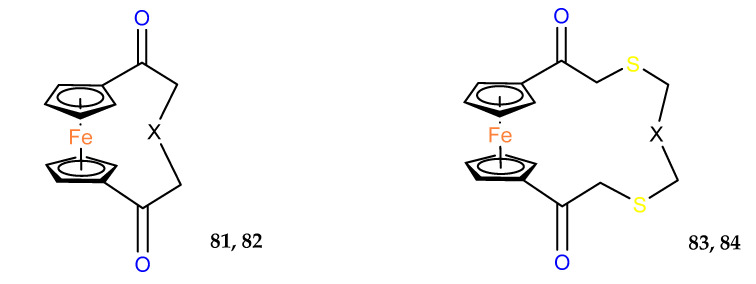					
Compound	X	Inhibition Ratio (%) (50 µg mL^−1^)			
		*A. solani*	*C. arachidicola*	*P. piricola*	*B. cinerrea*
**81**	S	40.0	40.9	68.9	85.7
**82**	NPh	64.0	50.0	88.5	100
**83**	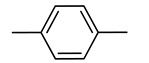	56.0	36.4	49.2	78.6
**84**	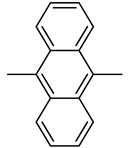	60.0	31.8	47.5	71.4

**Table 12:** Antimicrobial activity of citrate of **31** [50] and **85** [51].

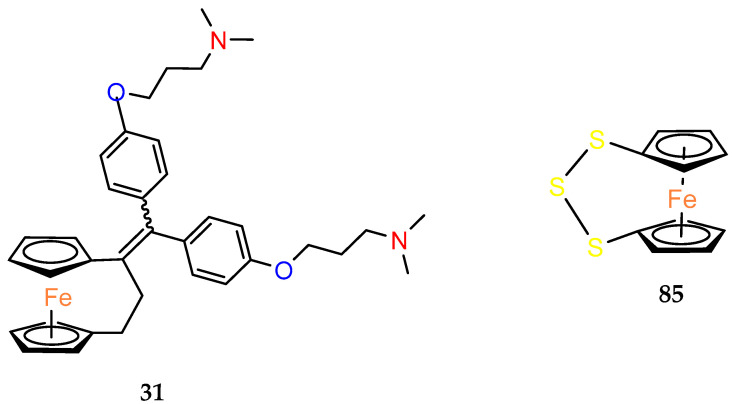				
Strains	MIC (μM)		Diameter of Zone Inhibition (mm)	
	Citrate of 31	85	85	
			30 (μg/disc)	200 (μg/disc)
*Listeria ivanovii*	12.5			
*Listeria monocytogenes*	16			
*Listeria monocytogenes*	8–16			
*Enterococcus faecalis*	20			
*Staphylococcus aureus*	8		00	14
*Escherichia coli*	100, 125	64	00	14
*Pseudomonas aeruginosa*	>5000		00	16
*Salmonella enterica*	625			
GM ^a^ (μM)	41.4			
HC_50_ ^b^ (μM)	22.4			
TI ^c^	0.5			
*Bacillus subtilis*		64	00	16
*Streptococcus β–haemolyticus*		64		
*Klebsiella species*		64	00	16
*Salmonella typhi*		128	09	17
*Shigella dysenteriae*			09	18
*Candida albicans*				10
*Aspergillus niger*				00
*Aspergillus flavus*				00

^a^ The observed geometric mean (GM) of the MICs of the compound against all bacterial strains. ^b^ HC_50_ is the minimal concentration that caused 50% hemolysis of red blood cells. ^c^ Therapeutic index is the ratio of the HC_50_ to the geometric mean of the MICs. Larger values mean greater cell selectivity.
